# Late-onset Lafora disease with prominent parkinsonism due to a rare mutation in *EPM2A*

**DOI:** 10.1212/NXG.0000000000000101

**Published:** 2016-08-16

**Authors:** David S. Lynch, Nicholas W. Wood, Henry Houlden

**Affiliations:** From the Department of Molecular Neuroscience (D.S.L., N.W.W., H.H.), UCL Institute of Neurology; and Neurogenetics Laboratory (H.H.), National Hospital for Neurology & Neurosurgery, Queen Square, London.

Lafora disease (LD) is an autosomal recessive form of progressive myoclonic epilepsy that is caused by mutations in *EPM2A*, encoding laforin, and *NHLRC1* (*EPM2B*), encoding malin.^1^ LD is classically described with onset in early teenage years. Patients develop myoclonus, epilepsy, visual hallucinations, and psychosis. Dementia is a prominent feature and often occurs in the late teenage years. LD typically progresses quickly, and patients become bedridden and dependent within 10 years of symptom onset, with life expectancy in the early 20s.^2,3^ Only a small number of late-onset cases of LD have been described. Even then, these so-called late-onset cases have typically presented in the 20s, with dementia occurring in the early 30s. We describe a patient with extremely late onset and extended survival with prominent parkinsonism due to a novel *EPM2A* variant.

The patient was a 53-year-old woman of South Asian ancestry who presented to the neurology clinic with a slowly progressive history of bradykinesia, upper and lower limb spasticity, and cognitive decline.

The patient had reached developmental milestones at appropriate ages and was cognitively normal as a young adult, graduating from university. Her parents were first cousins. She had a history of jerks affecting the upper and lower limbs, which had been present since her teenage years and were not troublesome. In her early 20s, she experienced several generalized seizures and was diagnosed with myoclonic epilepsy. The jerks and seizures ceased with sodium valproate treatment, and she eventually discontinued it.

The patient's sister was similarly affected with myoclonic jerks and seizures; later she developed memory and mobility difficulties. Her father had late-onset Parkinson disease.

When the patient's seizures recurred at age 43, sodium valproate was recommenced. On examination at age 45, she was diagnosed with mild cognitive impairment. The EEG taken was markedly abnormal with frequent generalized spike-and-wave complexes occurring every minute. Over the following 5 years, she continued to decline cognitively and stopped working. This was followed by the development of an extrapyramidal movement disorder with bradykinesia, cogwheel rigidity in the limbs, and difficulty mobilizing. On examination, she was found to have a dystonic facies with truncal and limb rigidity and marked bradykinesia. She had eyelid-opening apraxia, tonic deviation of the eyes upward, and impaired vertical eye movements. Self-injurious behavior developed, causing severe dermatitis artefacta to the nose, requiring reconstructive surgery.

A DATscan at age 53 demonstrated absent tracer uptake in the putamen bilaterally with severely reduced activity in the right caudate nucleus. Brain MRI revealed mild atrophy (figure). Skin and muscle biopsies were normal. Peripheral and cervical somatosensory evoked potentials revealed abnormally large amplitude cortical responses, suggesting cortical myoclonus.

**Figure:**
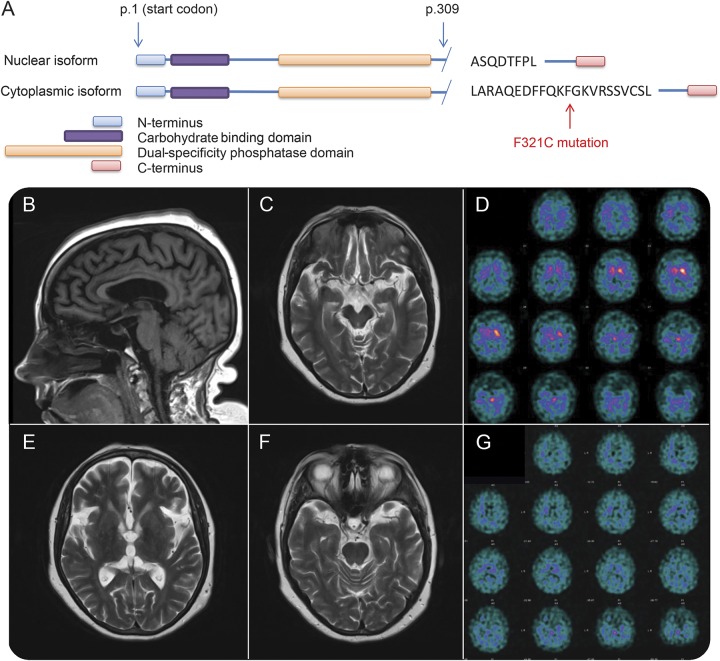
*EPM2A* gene and imaging appearance (A) Schematic diagram of the 2 major isoforms of laforin illustrating the F321C mutation, only affecting the cytoplasmic isoform of the protein. (B, C, E, F) MRI demonstrating only mild atrophy. The brainstem and basal ganglia are normal. (D) DATscan at age 53 shows severely reduced tracer uptake in the right caudate nucleus. (G) DATscan at age 59 shows severely reduced tracer uptake in the basal ganglia with increased background signal, indicating a profound dopaminergic deficit.

The patient's family reported that during sleep she would vocalize loudly and move her limbs, raising a suspicion of REM sleep behavioral disorder; however, overnight sleep study demonstrated catathrenia.

There was a short-lived response to levodopa (600 mg/d) and 5 mg selegiline with little response to dopamine agonists. Over the following years, she continued to decline and became increasingly dependent. She developed dysphagia and died at age 59 of a lower respiratory tract infection.

Exome sequencing was carried out, and we identified candidate genes by prioritizing rare (minor allele frequency <0.01), homozygous, and nonsynonymous coding variants. This identified 38 such variants, only one of which was found in a gene previously implicated in neurologic disease, *EPM2A*.

We identified a likely pathogenic homozygous mutation in *EPM2A* (c.962T>G p. F321C). The variant occurs at a highly conserved residue and has a frequency of 0.000008242 in the Exome Aggregation Consortium database.^4^ It was also found in the homozygous state in the patient's affected sister and heterozygous state in 2 unaffected siblings.

The *EPM2A* gene is alternatively spliced into at least 5 isoforms. Of the 2 major isoforms, one is 317 amino acids long and localizes to the nucleus while the other is 331 amino acids long and localizes to the rough endoplasmic reticulum.^5,6^ Almost all mutations in *EPM2A* have been found in the region of the gene common to both major isoforms.

The mutation found in this patient is predicted to affect only the cytoplasmic isoform of the laforin protein. This may explain the extremely late onset and slow progression of disease in our patient. From previous work, we can hypothesize that in our patient the cytoplasmic isoform has severely reduced the phosphatase activity^6^ while the nuclear isoform is unaffected.

Prominent parkinsonism has not previously been associated with LD and may have developed in the context of our patient's long survival. This case broadens the phenotype of LD and provides evidence of a genotype-phenotype correlation in the *EPM2A* gene.
